# Duodenal diverticulum perforation with pneumo-retroperitoneum: a case report of rare presentation

**DOI:** 10.1093/jscr/rjag085

**Published:** 2026-02-26

**Authors:** Yung-De Kuo, Chih-Ying Lu, Jinn-Rung Kuo, Chang-Yao Chu

**Affiliations:** School of Medicine, College of Medicine, National Sun Yat-sen University, No. 100, Shiquan 1st Rd., Sanmin Dist., Kaohsiung City 807378, Taiwan; Department of General surgery, Chi-Mei Medical Center, No. 901, Zhonghua Rd., Yongkang Dist., Tainan City 71004, Taiwan; Faculty Development Center, Chi-Mei Medical Center, No. 901, Zhonghua Rd., Yongkang Dist., Tainan City 71004, Taiwan; Department of Pathology, Chi-Mei Medical Center, No. 901, Zhonghua Rd., Yongkang Dist., Tainan City 71004, Taiwan

**Keywords:** duodenal diverticulum, perforation, retroperitoneal abscess, segmental duodenectomy, case report

## Abstract

Perforation of a duodenal diverticulum is rare but associated with high morbidity and mortality. Retroperitoneal perforations often present atypically, delaying diagnosis and management. We describe a 48-year-old woman presented to the hospital with abdominal pain after influenza A infection. Initial computed tomography suggested hollow viscus perforation, and she underwent laparoscopic lavage and drainage. Persistent bilious drainage and follow-up imaging revealed perforation of the third portion of the duodenum with retroperitoneal abscess. Exploratory laparotomy demonstrated a 2 × 2 cm perforated diverticulum at the inferior duodenal angle with surrounding necrosis. Segmental duodenectomy with duodenojejunostomy and feeding jejunostomy was performed. This case highlights the diagnostic difficulty of retroperitoneal duodenal diverticulum perforation and the importance of recognizing bilious drainage as an early warning sign. Conservative measures may be attempted in stable patients, but suspicion of ongoing leakage warrants timely definitive surgery. Early and comprehensive exploration may prevent delayed recognition and reduce morbidity.

## Introduction

Duodenal diverticula are common incidental findings, often detected on imaging or autopsy. Most remain asymptomatic; however, perforation is a rare but clinically significant complication [1–3].

Duodenal perforation—whether from diverticula, peptic ulcer disease, trauma, or iatrogenic injury—is a surgical emergency. It may present as free intraperitoneal or contained retroperitoneal perforation, with the latter often delaying diagnosis and complicating management [3–5]. Despite its rarity, duodenal perforation carries high mortality, emphasizing early recognition and prompt intervention [5, 6].

We report a case of perforated duodenal diverticulum at the inferior duodenal angle. Initially mistaken for a sealed microperforation, it progressed to a retroperitoneal abscess requiring segmental duodenectomy with duodenojejunostomy and feeding jejunostomy. This case highlights the diagnostic and surgical challenges of perforations in uncommon posterior duodenal sites [7–9]. Minimally invasive and endoscopic approaches have also been reported in selected cases [10].

## Case presentation

**Figure 1:**
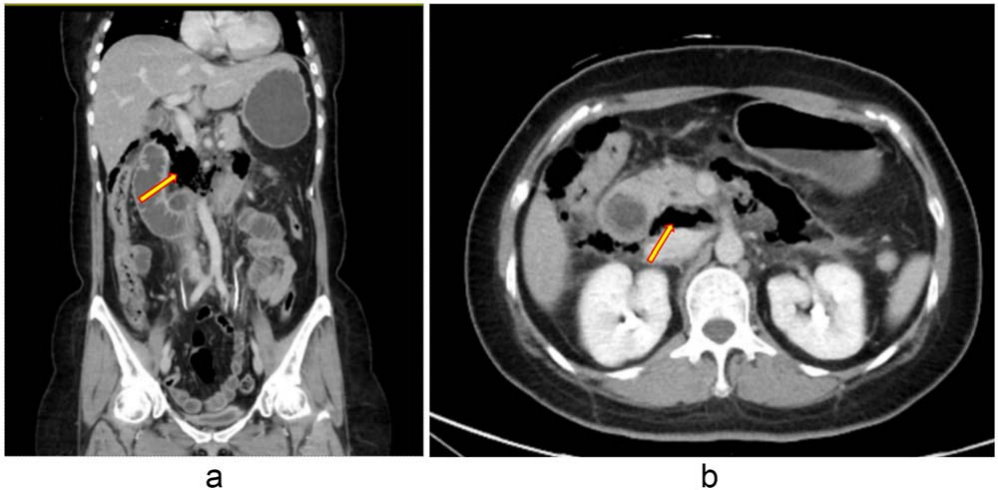
(a, b) Contrast-enhanced abdominal CT scan (26 August 2025). Imaging demonstrates retroperitoneal free air with suspicion of perforation in the retroperitoneal region.

A 48-year-old woman with adenomyosis and prior gynecologic surgeries was diagnosed with influenza A at a local hospital on 21 August 2025. On 26 August, she presented with 4 days of diffuse abdominal pain, fever, chills, and nausea. Computed tomography (CT) revealed intra-abdominal free air, suggesting hollow organ perforation (Fig. 1). Retrospective review of the initial CT demonstrated subtle indirect findings, including retroperitoneal fat stranding and mild wall thickening of the third portion of the duodenum, without definite localization of the perforation. She received intravenous piperacillin–tazobactam and underwent emergency surgery.

On admission, she was alert and hemodynamically stable (T: 36.7°C, HR: 98/min, RR: 18/min, BP: 137/76 mmHg). Physical examination showed mild right-sided tenderness without guarding. Labs revealed WBC 7850/μL (81% neutrophils), potassium 2.8 mmol/L, and CRP 400.1 mg/L.

**Figure 2:**
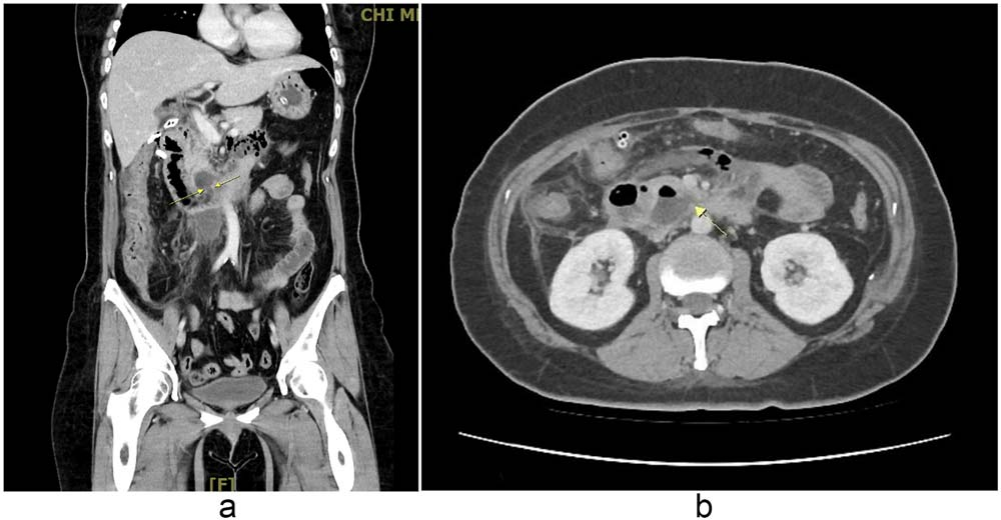
(a, b) Contrast-enhanced abdominal CT scan (coronal and axial views, 2 September 2025). Imaging demonstrates extraluminal air bubbles and a retroperitoneal abscess (arrow) adjacent to the third portion of the duodenum, consistent with duodenal perforation.

Laparoscopic lavage and abscess drainage yielded about 150 mL of turbid ascites in the subhepatic retroperitoneum without definite perforation, suggesting a sealed micro-perforation. The Estimated blood loss was 100 mL. Postoperatively, she received PCA, nasogastric drainage, parenteral nutrition, and antibiotics (cefazolin plus metronidazole, later flomoxef, then piperacillin–tazobactam). Persistent bilious drainage was noted. On postoperative day 2, she developed fever (38.4°C), leukocytosis (9790/μL), hypokalemia (2.7 mmol/L), and elevated CRP (154 mg/L). Despite therapy, bile leakage and right upper quadrant pain persisted. Follow-up CT on 2 September 2025 revealed probable perforation at the third portion of the duodenum with retroperitoneal abscess (Fig. 2). At her request, she was transferred to our hospital for further surgical management.

On arrival, physical examination showed diffuse tenderness with mild guarding, without rebound or costovertebral angle tenderness. Laboratory data confirmed leukocytosis and elevated CRP. With peritonitis impression, exploratory laparotomy was performed.

**Figure 3:**
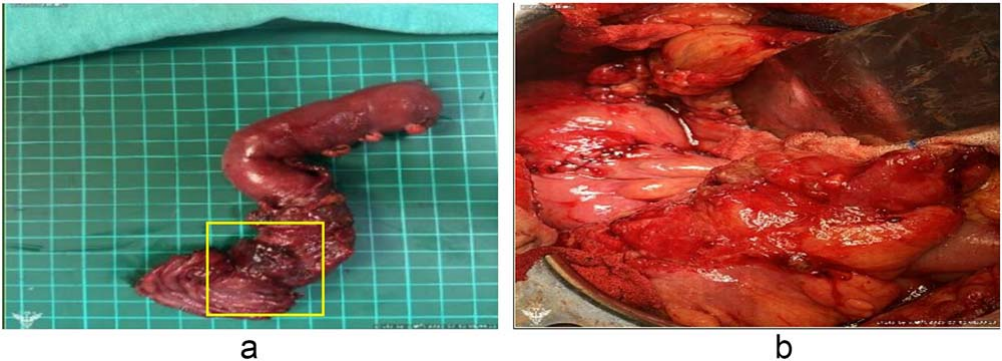
(a) The area of exposed mucosa represents the entire duodenal perforation. The yellow box highlights the perforation site. The apparent enlargement reflects *ex vivo* changes due to surgical dissection and traction. (b) Reconstruction following segmental duodenectomy. An end-to-side, hand-sewn, full-thickness duodenojejunostomy was performed using 4–0 monofilament absorbable sutures, followed by an additional outer layer of Lembert sutures.

Intraoperatively, a 2 × 2 cm perforation was found at the posterior wall of the inferior duodenal angle with surrounding pus and necrotic tissue (Fig. 3). A complete Kocher maneuver was performed to fully expose the posterior wall of the duodenum. Turbid whitish mucus appeared after the Kocher maneuver, and about 100 mL of turbid ascites were drained. After delineating the margins of the perforation based on gross intraoperative assessment, the involved duodenal segment containing the perforation was resected en bloc using a segmental resection approach, followed by end-to-side duodenojejunostomy and feeding jejunostomy. Two vacuum drains were placed (posterior and anterior to the anastomosis). The estimated blood loss was 800 mL.

**Figure 4:**
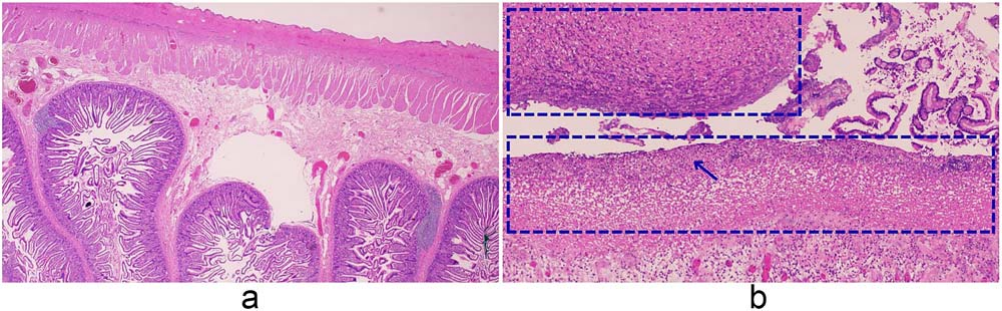
Histopathologic features of the small intestine and peritoneum. (a) Small intestine (2×), (b) small intestine with peritonitis (10×). (a) Low-power view of the small intestine showing preserved villous architecture and intact serosa. (b) Higher magnification (10×) demonstrating necrotic tissue within the dashed area, with dense neutrophilic infiltration (arrows), appearing as numerous small dark-staining cells, and purulent exudate on the serosal surface.

Pathology confirmed a perforated duodenal diverticulum at the inferior duodenal angle (Fig. 4). The 15 × 5.5 cm specimen contained a 5.5 × 4 cm reddish lesion with attached omentum and appendix. Microscopically, purulent exudates with acute and chronic inflammation were consistent with perforation, and both omentum and appendix showed suppurative peritonitis.

The patient continued postoperative antibiotic therapy for infection control. White blood cell counts and C-reactive protein levels gradually normalized postoperatively, and the bilious drainage tube was subsequently removed. She showed gradual clinical improvement and was successfully discharged 23 days after surgery.

## Discussion

Duodenal diverticula are relatively common, appearing in up to 22% of autopsy and imaging studies, though only 1%–5% become symptomatic. Perforation is rare but carries a mortality rate of up to 30% [2, 3, 5]. Most diverticula occur in the second portion, while those at the inferior duodenal angle of the third portion are uncommon. Posterior perforations frequently extend retroperitoneally, leading to vague symptoms and delayed diagnosis [4, 7]. In this case, such a retroperitoneal perforation caused diagnostic difficulty and required extensive resection with reconstruction due to necrosis.

CT remains the primary diagnostic tool, typically demonstrating free intra- or retroperitoneal air; however, small or sealed perforations may obscure localization [1, 2]. In this patient, no defect was found during initial laparoscopic lavage despite Kocherization, underscoring the importance of systematic exploration of the third and fourth portions. Retrograde access via the caudal side of the transverse mesocolon and Treitz ligament can enhance exposure when the cranial approach is limited [7].

Retroperitoneal duodenal perforation may mimic acute pancreatitis, periappendiceal abscess, or colonic diverticular perforation, leading to diagnostic delay. However, persistent bilious drainage is not expected in these conditions and strongly suggests an ongoing duodenal leak. From a pathophysiological standpoint, continuous bile flow into the second and third portions of the duodenum explains sustained bile-rich drainage in posterior distal duodenal perforation, even in the absence of marked intraperitoneal contamination.

Management of perforated duodenal diverticula remains controversial. In the present case, initial CT demonstrated only nonspecific retroperitoneal free air, whereas follow-up imaging revealed localized extraluminal air and an organized retroperitoneal abscess adjacent to the third portion of the duodenum, a pattern consistent with reported delayed abscess formation in contained retroperitoneal perforations [3]. Conservative or minimally invasive options, including drainage and endoscopic decompression, may succeed in selected retroperitoneal perforations [9]. Such approaches are appropriate when the perforation is contained, and the patient remains hemodynamically stable [8]. However, deterioration, uncontrolled infection, or diffuse peritonitis necessitate surgery [3, 6]. Our patient exemplified this course: initial laparoscopic lavage failed, leading to a retroperitoneal abscess that required definitive operation. Early and comprehensive exploration of all duodenal segments is critical, as large defects rarely seal spontaneously, and timely surgery reduces morbidity [5].

Although disease-specific guidelines for spontaneous duodenal diverticulum perforation are lacking, general management principles for gastrointestinal perforations have been proposed. The European Society of Gastrointestinal Endoscopy position statement emphasizes patient stability, extent of peritoneal contamination, and response to conservative measures as key determinants for escalation to surgical intervention [11].

Intraoperative findings demonstrated a 2 × 2 cm posterior perforation at the inferior duodenal angle with surrounding pus and necrosis, consistent with a perforated duodenal diverticulum. Absence of an intact diverticular wall on histopathology is common, as necrosis and inflammation often obliterate structural landmarks [3, 7]. Similar reports, including those describing endoscopic drainage or bezoar removal, show that atypical retroperitoneal perforations can mimic other intra-abdominal diseases, delaying diagnosis [10]. Such variability underscores the importance of intraoperative confirmation and individualized surgical planning [8].

The failure of initial laparoscopic drainage in our patient was likely related to the posterior location of the perforation in the third portion of the duodenum, the relatively large defect (2 × 2 cm), and the presence of surrounding necrotic tissue, which limited the potential for spontaneous sealing and ongoing retroperitoneal contamination. Although simple repair or diverticulectomy has been reported in selected cases, segmental duodenectomy was favored in our patient due to the extent of necrosis and the inability to achieve a secure closure with viable tissue, allowing definitive source control and tension-free reconstruction.

Perforation of the third portion of the duodenum poses specific anatomical challenges due to its retroperitoneal location and proximity to the pancreatic head, often resulting in subtle clinical presentation and delayed diagnosis. As reported in previous studies [7, 9], exposure and definitive management of distal duodenal perforations are technically demanding, further supporting the need for individualized surgical strategies in this rare location.

Retroperitoneal duodenal perforations are frequently deceptive, lacking classic peritoneal signs and delaying recognition [4, 6]. Persistent bilious drainage should raise suspicion of duodenal leakage. When large defects or necrosis are present, definitive surgery such as segmental duodenectomy with duodenojejunostomy and feeding jejunostomy may be required [7, 9]. These findings support the literature emphasizing early recognition, vigilant monitoring, and timely surgical intervention to improve outcomes [3, 5]. Long-term follow-up after segmental duodenectomy should include endoscopic surveillance to assess anastomotic patency, nutritional monitoring, and attention to symptoms suggestive of biliopancreatic reflux or anastomotic stenosis.

## Conclusion

Duodenal diverticular perforation, particularly in the third portion with retroperitoneal extension, is a rare but serious surgical challenge. This case highlights the subtle course of retroperitoneal perforation, the value of persistent bilious drainage, and the need for thorough exploration. When necrosis is extensive, segmental duodenectomy with duodenojejunostomy provides definitive management. Early recognition and timely intervention are crucial to improve outcomes in this uncommon yet life-threatening condition.
